# Diet and ovarian cancer risk: a case–control study in China

**DOI:** 10.1038/sj.bjc.6600085

**Published:** 2002-03-04

**Authors:** M Zhang, Z Y Yang, C W Binns, A H Lee

**Affiliations:** School of Public Health, Curtin University of Technology, GPO Box U1987, Perth, WA 6845, Australia; Department of Gynecological Oncology, Zhejiang Cancer Hospital, Hangzhou, China

**Keywords:** case–control study, diet, ovarian cancer, risk factors

## Abstract

This case–control study, conducted in Zhejiang, China during 1999–2000, investigated whether dietary factors have an aetiological association with ovarian cancer. Cases were 254 patients with histologically confirmed epithelial ovary cancer. The 652 controls comprised 340 hospital visitors, 261 non-neoplasm hospital outpatients without long-term diet modifications and 51 women recruited from the community. A validated food frequency questionnaire was used to measure the habitual diet of cases and controls. The risks of ovarian cancer for the dietary factors were assessed by adjusted odds ratios based on multivariate logistic regression analysis, accounting for potential confounding demographic, lifestyle, familial factors and hormonal status, family ovarian cancer history and total energy intake. The ovarian cancer risk declined with increasing consumption of vegetables and fruits but *vice versa* with high intakes of animal fat and salted vegetables. The adjusted upper quartile odds ratio compared to the lower quartile was 0.24 (0.1–0.5) for vegetables, 0.36 (0.2–0.7) for fruits, 4.6 (2.2–9.3) for animal fat and 3.4 (2.0–5.8) for preserved (salted) vegetables with significant dose-response relationship. The risk of ovarian cancer also appeared to increase for those women preferring fat, fried, cured and smoked food.

*British Journal of Cancer* (2002) **86**, 712–717. DOI: 10.1038/sj/bjc/6600085 www.bjcancer.com

© 2002 Cancer Research UK

## 

Ovarian cancer is the seventh most common cancer in women and the leading cause of death among gynecological cancers (Kristensen and Trope, 1997). However, little is known of its aetiological factors (World Cancer Research Fund, 1997). Dietary factors, especially vegetables, fruits and fat intake, have been suggested to influence ovarian cancer risk (Snowdon, 1985; La Vecchia *et al*, 1987; Shu *et al*, 1989; Tzonou *et al*, 1993; Risch *et al*, 1994; Kushi *et al*, 1999; Parazzini *et al*, 2000). Shu *et al* (1989) found a significant positive dose-response relationship between intake of fat from animal sources and ovarian cancer, and a somewhat protective effect from vegetables, in a case–control study in China. The aim of this case–control study is to further assess the association between dietary factors and epithelial ovarian cancer, which accounts for more than 90% of all ovarian malignancies (Cotran *et al*, 1999).

## MATERIALS AND METHODS

### Study design and participants

A case–control study was conducted in Hangzhou, China, between July 1999 and June 2000. Two hundred and fifty-five hospital patients with epithelial carcinoma of the ovary were identified. To ensure complete ascertainment of cases, all medical records and laboratory pathology reports were reviewed during the period of the study. Pathological diagnoses were based on the International Histological Classification of Ovarian Tumours (Cotran *et al*, 1999; Underwood, 2000). A total of 254 patients (non-response rate 0.04%) participated in the study. Inclusion criteria for cases were defined to be women under 75 years of age, who were residents (at least 10 years residence in Zhejiang province) and who had been histopathologically diagnosed with epithelial ovarian cancer in the past 3 years. Most of the cases (75.2%) were recent patients interviewed within 12 months from diagnosis.

During the same period of recruitment 652 controls were also interviewed. Women recruited for controls were matched with cases by age and geographical area. Exclusion criteria for the controls were neoplasm, long-term dietary modification or who had bilateral oophorectomy. A total of 601 women were recruited from the same hospitals where the cases were identified. The control group consisted of 340 hospital visitors (15 women declined to be interviewed, non-response rate 4.4%) and 261 outpatients (non-response rate 1.2%).

To control for bias in selecting the hospital controls, consulting rooms for outpatients and ward numbers for healthy women were chosen randomly. If no suitable subjects were found in the chosen room/ward, the adjacent room/ward was used instead. All the outpatients recruited had only minor gynecological diseases (84.7% had vulvitis, vaginitis or cervicitis, 6.5% diagnosed with urethritis and 5.7% had menopausal symptoms).

An additional sample of 51 community women (non-response rate 7.8%) was recruited from nine different districts of Hangzhou. The project was approved by the appropriate hospital authorities and the human research ethics committee of the researchers' institution.

### Food frequency questionnaire

A structured questionnaire, available from the authors upon request, was used to collect the required information on diet, lifestyle and factors relevant to hormonal status. The quantitative Food Frequency Questionnaire (FFQ) component on habitual diet was modified from a dietary questionnaire (Ji *et al*, 1998) used for studying cancers in Shanghai, in order to ensure cultural relevance. Additional questions were taken from the Hawaii Cancer Research Survey (Thompson and Byers, 1994), the Australian Health Survey 1995 (ABS, 1995), and the USA food survey 1992 (NIS, 1992). The face validity and content validity of the FFQ were verified in a preliminary test. In a subsequent test–retest, the correlation coefficient ranged between 0.43 and 0.96 for mean food group consumption while the Kappa statistic ranged between 0.27 and 0.89 for eating habits, confirming the suitability of the FFQ to measure habitual diet.

The FFQ contained questions on 120 food items, which included all of the foods in the usual diet of Zhejiang residents. Information was also sought on the quantities of each food consumed per meal as well as cooking methods used and vitamins or mineral supplements taken. Food consumption was based on habitual diet and a ‘reference’ recall period was set at 5 years prior to diagnosis (cases) or interview (controls). The food frequency variables were categorised into never or hardly ever, once a month, 2–3 times a month, once a week, 2–3 times a week, 4–6 times a week, once a day and ⩾2 times a day. The quantitative variables were measured in terms of the most common Chinese measure, the *liang* (equivalent to 50 g).

### Interviews

Data were collected by face-to-face interview after obtaining their consent. The interviews usually took between 40 and 50 min. Since Chinese women are typically responsible for buying raw foods and cooking for the household, the participants were able to provide information on the frequency and quantity of each food consumed per meal. Standard sized reference containers were provided to assist respondents to accurately estimate the weight of food items.

For the cases, 226 women (89%) were either inpatients or at hospitals for follow-up treatment, 25 women (10%) were interviewed at their homes, while three women (1%) completed their interviews at their work places. The 51 community women were interviewed at their homes, community areas near their homes or their work places.

### Data management

All data were reviewed for completeness at the end of each interview. The frequency and quantity variables were converted into the quantities of foods consumed. Adjustments were made for the edible portions of foods, cooking methods used, seasonal factors, and market availability. The total energy intake from the 119 food items was calculated based on the *Table of Food Components* (Institute of Nutrition and Food Hygiene, 1999), except the conversion of coffee was based on US data (First Databank Incorporate, 1999).

### Statistical analysis

The data were coded and analysed using the SPSS package (SPSS, 2000). The mean food consumption (kg per year) was tabulated separately for case and control groups. To control for hospital bias, data from the three control groups were initially compared to assess any differences. As little differences were found between them, the control groups were then combined prior to comparison with the cases. Univariate logistic regression was used to screen out potentially significant variables for subsequent multivariate analysis. To minimise survival bias, data for the 191 recent patients and data for all cases were analysed separately.

The quantities consumed for the various food groups and the eating habits categorical variables were tested for linear trends in ovarian cancer risk. The quantity variables were also divided into quartiles based on the corresponding distribution in the controls. Odds ratios (OR) and their 95% confidence intervals (CI) were computed, using unconditional multivariate logistic regression models. Food intake and eating habits were assessed separately. Within each setting, all variables were simultaneously considered in a single model to allow for possible reciprocal confounding effects. Both regression equations included terms for age at interview, education, living area, BMI (5 years ago), smoking, alcohol and tea drinking, family income (*yuan* per month), marital and menopause status, parity (full-term pregnancy), tubal ligation, oral contraceptive use, physical activity, family history of ovarian cancer and total energy intake.

## RESULTS

There were no significant differences between the two hospital control groups with respect to food groups, total energy intake and other variables, with the exception of dairy products and vegetable oil (which had little association with ovarian cancer risk). The mean intakes of food groups by the community controls were also similar but they consumed less salted vegetables and animal fat than the hospital controls. Data from all three control groups were then combined to form a single control group in subsequent analyses. Results from recent patients and all cases were also similar (data not provided for brevity but were available upon request). Therefore, we report the combined results of all cases below.

There were no differences in mean age at interview, locality (urban or rural areas), education, BMI (5 years ago), smoking, alcohol consumption, marital and menopause status, and tubal ligation between cases and controls. Compared to controls, patients with epithelial ovarian cancer tended to have a higher family income, less oral contraceptive use, lower parities, and less physical activity. More of them had apparent family susceptibility. Table 1

**Table 1:**
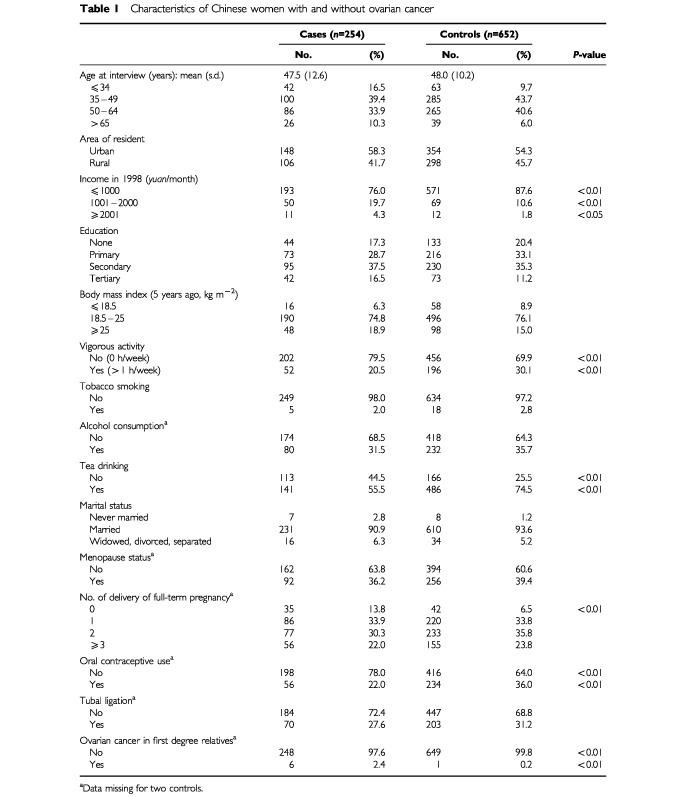
Characteristics of Chinese women with and without ovarian cancer

contrasts the sample characteristics of women with and without ovarian cancer.

Table 2

**Table 2:**
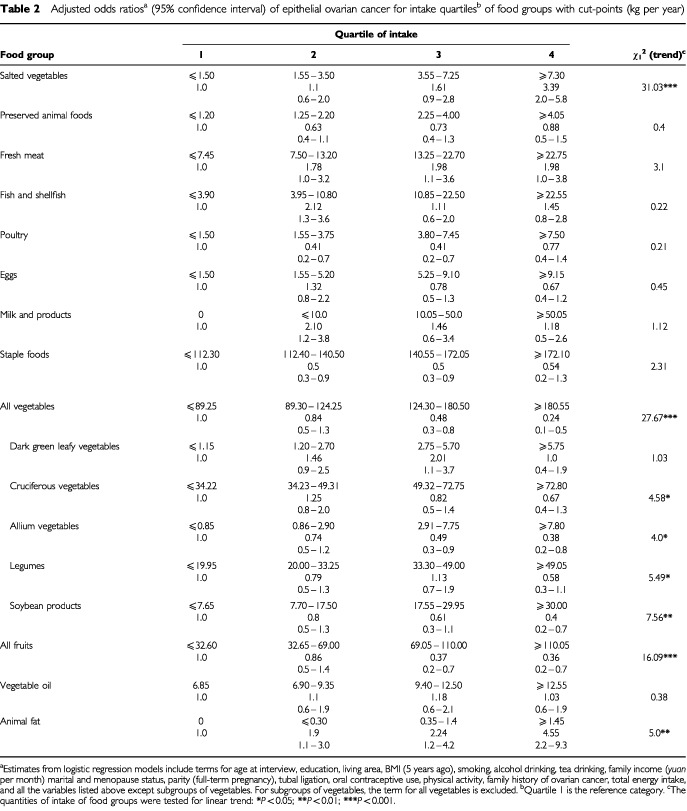
Adjusted odds ratios^a^ (95% confidence interval) of epithelial ovarian cancer for intake quartiles^b^ of food groups with cut-points (kg per year)

gives the odds ratios of ovarian cancer according to intake quartile of food groups, together with results of linear trend tests for the corresponding continuous variables. The risk tended to decline with increasing consumption of vegetables and fruits. Intakes of animal fat and preserved vegetables were associated with an elevated risk of ovarian cancer. The adjusted OR's of the upper quartile, relative to the lower quartile, were 0.24 for vegetables, 0.36 for fruits, 4.6 for animal fat and 3.4 for preserved vegetables, with significant dose-response relationships. Higher intakes of fresh meat appeared to be positively associated with ovarian cancer, whereas the intake of staple foods seemed to offer some protection, but both chi-square tests of linear trend were insignificant. Among the subgroups of vegetables, the estimated OR's were 0.38 for allium vegetables and 0.4 for soybean products. No significant association was evident with respect to preserved animal foods, fresh fish, poultry, eggs, dairy products and plant oil.

Table 3

**Table 3:**
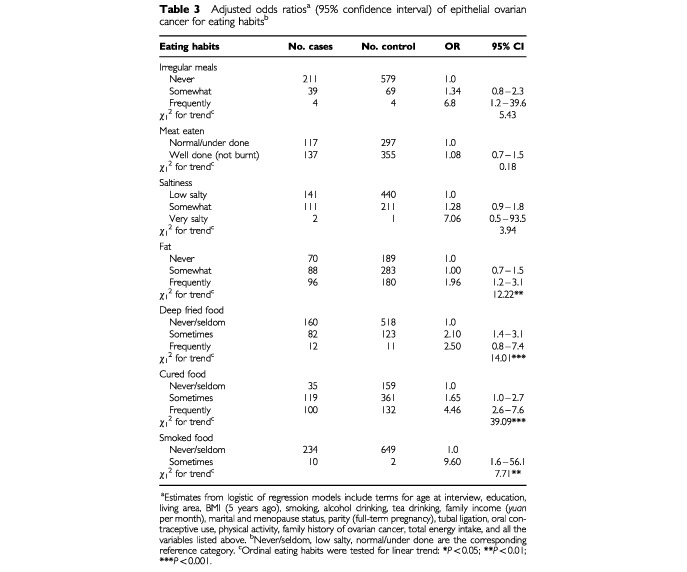
Adjusted odds ratios^a^ (95% confidence interval) of epithelial ovarian cancer for eating habits^b^

gives the OR for eating habits, the frequency of which were also subjected to linear trend testing. The risk of ovarian cancer appeared to increase with preference to fat, fried, cured and smoked foods. The estimated OR's of frequent intake compared to the never/seldom category were 1.96 for fat, 2.50 for fried food, 4.46 for cured food and 9.60 for smoked food, with all the corresponding trends being significant at the 5% level.

## DISCUSSION

Results from the present study supported the previous findings by Shu *et al* (1989) conducted in a similar region in China, especially that an elevated risk was associated with an increased consumption of animal fat. To the best of our knowledge, the present study is the first to investigate the effect of preserved foods with respect to ovarian cancer risk. The protective effects of fresh fruits and vegetables are consistent with other case–control studies on epithelial ovarian cancer conducted elsewhere.

Within the vegetables group, the subgroups of allium vegetables and soybean products had appreciable inverse associations with ovarian cancer. The mechanism of protective effects from vegetables and fruits may be partly due to the presence of antioxidants (Hollman and Katan, 1999; Ekstrom *et al*, 2000) or may be attributed to the phytochemicals present (Steinmetz and Potter, 1991).

An elevated risk of ovarian cancer due to an increased consumption of saturated fat had been reported previously (La Vecchia *et al*, 1987; Risch *et al*, 1994). Conversion data were not available on saturated and unsaturated fat in Chinese foods. However, we measured intake of animal fat (lard) as a proxy for saturated fat and obtained a similar positive result.

A new finding was the positive association between salted vegetables and ovarian cancer. Salted foods have been reported to increase the risk of stomach cancer and esophagus cancer in several Chinese case–control studies (Gao *et al*, 1994; Ji *et al*, 1995, 1998). Salted foods tend to contain large quantities of nitrites and nitrates that may serve as substrates for endogenous formation of *N-*nitroso compounds (Armstrong and Doll, 1975; O'Neill *et al*, 1991). However, we found preserved animal foods, which are rich in nitrites and nitrosamines (Armstrong and Doll, 1975; O'Neill *et al*, 1991), induced no significant change in risk. Unlike salted vegetables, preserved animal foods are relatively expensive in China and their consumption levels are low. The minimal impact of preserved animal foods was also documented in a previous study on esophageal, stomach and pancreatic cancers (Ji *et al*, 1998).

In a 13-year follow-up of 142 857 Japanese women, Hirayama (1981) showed that high meat intake could lead to an elevated risk of ovarian cancer. Our study suggested a positive association for meat consumption but the trend was not significant. The association was apparently related to fat consumption since over 90% of meat intake in this study was derived from pork. Eggs and milk were not associated with ovarian cancer risk. Although intake of eggs and milk by Chinese women has increased over the past two decades, it is still relatively low compared with occidental women (Guo, 1997).

This study also assessed the link between dietary habits and ovarian cancer. Frequent intake of fat and fried foods has led to elevated risks of the cancer. Fried food was related to fat consumption because fat, especially animal fat, was typically used in the process of deep-frying. In a 20-year follow-up of 16 190 females, Snowdon (1985) found a positive association between consumption of fried foods and fatal ovarian cancer. Similarly, a preference for eating cured and smoked foods could be confounded by a higher intake of salted vegetables.

Several issues should be considered when interpreting the findings. A major feature of this study is that extensive information on food intake and dietary patterns was obtained using a validated and reliable instrument specifically targeted on adult Chinese women. Furthermore, mean energy intake by the controls, as calculated from our FFQ, was 2399 Kcal per day (Zhang *et al*, 2002), which was comparable to the 2433 Kcal per day consumed by Zhejiang residents (Ge *et al*, 1995).

Case–control studies are subjected to several potential biases. Firstly, in our study, selection bias appeared to be minimal in view of the low refusal rate. The majority of cases were recently diagnosed and the recruitment procedure used was designed to ascertain all cases. Recall bias was minimised by reporting habitual diet and by using a ‘reference’ recall period. Although the small number of community controls consumed less salted vegetables and animal fat than the hospital controls, their exclusion did not influence the findings of this study.

In conclusion, our results suggest that a diet rich in fresh vegetables and fruits, but less animal fat, salted vegetables, fried, cured and smoked food, contribute to a lower risk of ovarian cancer.
